# The intertwining between lead and ethanol in the model organism *Caenorhabditis elegans*

**DOI:** 10.3389/ftox.2022.991787

**Published:** 2022-09-20

**Authors:** P. A. Albrecht, L .E. Fernandez-Hubeid, R. Deza-Ponzio, M. B. Virgolini

**Affiliations:** ^1^ Departamento de Farmacología Otto Orsingher, Facultad de Ciencias Químicas, Universidad Nacional de Córdoba, Córdoba, Argentina; ^2^ Instituto de Farmacología Experimental de Córdoba-Consejo Nacional de Investigaciones Científicas y Técnicas (IFEC-CONICET), Facultad de Ciencias Químicas, Universidad Nacional de Córdoba, Córdoba, Argentina

**Keywords:** lead-exposure, ethanol, dopamine, *Caenorabditis elegans*, rats

## Abstract

*Caenorhabditis elegans (C. elegans)* is a model organism widely used to evaluate the mechanistic aspects of toxicants with the potential to predict responses comparable to those of mammals. We report here the consequences of developmental lead (Pb) exposure on behavioral responses to ethanol (EtOH) in *C. elegans*. In addition, we present data on morphological alterations in the dopamine (DA) synapse and DA-dependent behaviors aimed to dissect the neurobiological mechanisms that underlie the relationship between these neurotoxicants. Finally, the escalation to superior animals that parallels the observed effects in both experimental models with references to EtOH metabolism and oxidative stress is also discussed. Overall, the literature revised here underpins the usefulness of *C. elegans* to evidence behavioral responses to a combination of neurotoxicants in mechanistic-orientated studies.

## Introduction

As a model organism first described by Brenner in 1973 (Brenner, 1973), *Caenorhabditis elegans (C. elegans)* is a suitable model to study the neurobiological basis of toxicity. This small living invertebrate has provided invaluable evidence for the neurotoxic mechanisms of several elements, including lead (Pb) (Chen et al., 2013; Jiang et al., 2016; Soares et al., 2017). Additionally, many responses to ethanol (EtOH), not only in movement-related behaviors, but also in other parameters, have revealed its potential to study the neurobiological bases of drugs of abuse (Grotewiel and Bettinger, 2015; Engleman et al., 2016; Katner et al., 2019).

Thus, in the present review, we intended to provide evidence to support the role of *C. elegans* as a prominent organism for the study of the mechanisms underpinning the adverse effects of Pb and the specific factors involved in the vulnerability to EtOH addiction (Bettinger and Davies, 2014; Engleman et al., 2016; Khanh, 2018). Furthermore, its potential translational value from studies in mammals was also considered.

### *C. elegans* as a model in toxicological studies

This transparent, free-living nematode has the potential to predict responses comparable to mammals (Anderson et al., 2004; Hunt et al., 2018; Meneely et al., 2019), without the ethical issues involved in higher animal experimentation (Casey et al., 2015). The simplicity of the cell lineage and the existence of a variety of transgenic animals determine that *C. elegans* has become a model widely used in toxicity tests to evaluate the mechanistic aspects of a myriad of substances (Hunt et al., 2020). Its well-described nervous system allows for studying the cellular mechanisms that underlie neurodegeneration (Caldwell et al., 2020), including Parkinson’s disease (Maulik et al., 2017; Cooper and van Raamsdonk, 2018; Gaeta et al., 2019) and Alzheimer’s diseases (Paul et al., 2020; Y. Wu and Luo, 2005), along with the pesticides and metals possibly involved in their etiology or progression (Gonzalez-Hunt et al., 2014; Soares et al., 2017; Sedensky and Morgan, 2018; Martins et al., 2022). To this end, manganese, mercury, and Pb in particular are the metals considered the most potentially hazardous to human health (Avila et al., 2016; Caito and Aschner, 2016; Lu et al., 2018; Akinyemi et al., 2019).

### Lead

This toxic, persistent, and non-essential metal that accumulates in the environment and living organisms induces damage to all systems, including the central nervous system (Virgolini and Aschner, 2021). Early-life Pb exposure may cause an imprint that can be evident later in life or in response to a variety of challenges (Silbergeld, 1992; Mitra et al., 2017; Vorvolakos et al., 2016; Virgolini et al., 2019). At the functional level, many neurotransmitters have been studied after Pb exposure (Xing et al., 2009a; Xing et al., 2009b; Sudama et al., 2013), with the deleterious effects on DAergic neurons among the best described in different experimental models (Zuch et al., 1998; NourEddine et al., 2005; Szczerbak et al., 2007).

Several studies show that short periods of Pb exposure during adulthood (less than 12 h) resulted in decreased locomotion (Boyd et al., 2003; Wang and Xing, 2009), changes in movement patterns (Wang and Xing, 2008), or reduced feeding behavior (Anderson et al., 2001, 2004). Other reports indicate that acute exposure to this metal decreased memory (Ye et al., 2008) or associative learning in a thermotaxis assay (Zhang et al., 2010), effects that are reversed by the pretreatment with antioxidant agents such as dimethyl sulfoxide (DMSO) or N-acetylcysteine (NAC) (Wu et al., 2012). These results suggest that oxidative stress could be involved in the mechanism of neurotoxicity exerted by this metal (Virgolini and Aschner, 2021). Importantly, Guo et al. observed defects in the reproductive capacity of the worm (decreased egg-laying numbers and lengthened generation of progeny), alterations present across all stages, with developing larvae being more vulnerable to Pb than young adults (Guo et al., 2009).

Regarding long exposures, Tiwari et al. inform alterations not only in locomotor activity, but also in the growth pattern in nematodes exposed to sublethal Pb concentrations (3 μm, 15 μm, or 30 μm Pb) for 24 h. In these studies, they reported dose-dependent alterations in reverse movements, a decrease in body length, and an increase in the peristaltic velocity (Tiwari et al., 2020). In this line, other researchers reported reduced body bends, head movements, and reverse movements after Pb exposure, mitigated by pretreatment with selenium (Li et al., 2013).

In terms of chronic treatments, Wang and Yang (2007) showed that sustained exposure to Pb for 3 days induces multiple dose-dependent biological effects in the nematode, including shortened half-life, decreased body size, reproductive abnormalities, and defects in the function of the nervous and muscular systems, with many effects transferable to the progeny (Wang and Yang, 2007). In the same line, Yu et al. reported growth inhibition and changes in movement patterns, which turned out to be more evident in the second generation, data that reinforces the importance of the developmental stage at which Pb exposure occurs (Yu et al., 2013). Interestingly, Pb-induced changes in growth, feeding, and reproduction persisted for up to four generations and may even be more noticeable in the last one (Yu et al., 2016). Moreover, transgenerational alterations in parameters such as growth rate, motility, feeding, and/or reproduction have been reported when Pb exposure occurred during stages spanning gonad and egg development (Wang and Yang, 2007; Yu et al., 2013, 2016).

Regarding behavioral alterations, Sun et al. (2016) reported a decrease in locomotor activity as well as a shorter lifespan in worms exposed for 36 h to 8.5 μM Pb(NO_3_)_2_, from the L1 stage to the adult L4 (Sun et al., 2016). Interestingly, Monteiro et al. (2014) observed the opposite phenomenon: exposure to moderate to high Pb doses for 4 days reduced larval movement and reproduction (Monteiro et al., 2014). However, after low concentrations (less than 0.5 µm), a stimulatory effect on reproduction and growth was observed, possibly due to survival or a dispersal strategy manifested in a stressful environment (Roh et al., 2006; Monteiro et al., 2014). This behavior may be indicative of hormesis, a phenomenon that occurs when low concentrations of a toxicant elicit an adaptive response, which is stimulant in this case, protecting the organism against subsequent exposures to higher doses of the same pollutant (Wang and Xing, 2009; Zhao and Wang, 2012).

Overall, although still scarce, the reported evidence demonstrates that *C. elegans* is a suitable model to study the adverse effects of Pb exposure in immature and adult organisms in terms of developmental neurotoxicity (Ruszkiewicz et al., 2018) and transgenerational studies (Zhao et al., 2022).

### Ethanol

Ethanol (EtOH) is an easily accessible drug of abuse that induces biphasic responses in living organisms depending on its metabolism and effects on the CNS (Pohorecky, 1977; Hendler et al., 2013; Virgolini and Pautassi, 2022). In humans, acute exposure to low EtOH doses induces hyperactivity and euphoria, mild doses are anxiolytic, while high exposures cause impaired coordination and balance, sedation, and even death (Zhu et al., 2014). Although these behaviors can be a sensitive indicator of toxicity, they are a complex phenomenon hard to quantify in higher organisms. Based on these considerations, *C. elegans* allows the assessment of simple behaviors that are shown as alterations in locomotion and measurable as changes in speed or direction closely related to behaviors observed in humans. It has been demonstrated that worms exposed to EtOH evidenced initial hyperactivity followed by immobility, which is reversed when EtOH exposure ceased (Wu et al., 2019). Similarly, low EtOH concentrations (17–52 mM) produce hyperactivity, whereas amounts between 100 and 400 mM decrease motility (Dhawan et al., 1999; Morgan and Sedensky, 1995; reviewed in Scholz and Mustard, 2013). In addition, acute exposure to this drug induces a dose-dependent depression in locomotion and egg-laying behavior at comparable internal EtOH concentrations known to induce intoxication in humans and other mammals (Alaimo et al., 2012).

Furthermore, this nematode shows two well-described behaviors in vertebrates known as tolerance and sensitization that are distinctive in humans in response to excessive consumption of psychoactive substances (Lee et al., 2009; Bettinger and Davies, 2014; Grotewiel and Bettinger, 2015). Interestingly, continuous exposure to EtOH generates the development of a behavioral phenomenon representative of neuronal plasticity called “acute functional tolerance” (AFT) (Davies and McIntire, 2004), first described in rodents (LeBlanc et al., 1975). This behavior is evident when worms recover part of their mobility after the decrease in the speed of movement or locomotion on a solid agar surface as a consequence of the exposure to high EtOH concentrations (Raabe et al., 2014; Davies et al., 2015). Notably, both neuronal plasticity and the mechanisms underlying AFT are considered a compensatory response to the environmental insult elicited by the actions of EtOH (Raabe et al., 2014).

In addition to the above-described behavior, *C. elegans* exhibits the fundamental features of EtOH withdrawal symptoms reported in higher animals, including humans (Scott et al., 2017). Several behaviors modified by EtOH withdrawal can be partially or fully reversed by re-exposure to a low EtOH dose (Scott et al., 2017). In this regard, McIntire (2010) demonstrated that during EtOH abstinence the worms showed altered posture and impaired ability to direct themselves towards food (McIntire, 2010). These and others researchers (Crowder, 2004) also reported the involvement of slo-1, a highly conserved gene encoding for the calcium- and voltage-gated long-conductance K channel (also called BK potassium channel or SLO-1, homologous to the same proteins in humans). Interestingly many other responses associated with EtOH also appear to be modulated by the expression of this gene (Davies et al., 2003; Scott et al., 2017).

Finally, in a recent work, Sterken et al. (2021) studied the time-course transcriptional modifications of EtOH exposure. They reported that 400 mM EtOH induced transcriptional profiles in many genes at long exposure periods. Oppositely, short exposures to EtOH (up to 2 h) induced the expression of enzymes involved in its metabolism, particularly ADH, the enzyme involved in EtOH oxidation to acetaldehyde. On the other side, longer exposures (8 h or more) had much more profound effects on the transcriptome and genes involved in neuronal function, lipid microenvironment, and physiological responses to EtOH, including direct targets of this drug (Sterken et al., 2021).

Overall, this evidence demonstrates that despite some limitations, *C. elegans* is a powerful tool for identifying critical developmental periods in which EtOH could cause subsequent delays (Lin et al., 2013). In addition, this model organism permits the assessment of simple behaviors and the identification of epigenetic factors, genes, and/or proteins that regulate EtOH-related effects that may be potential therapeutic targets for the treatment of alcohol use disorders (AUD) (Khanh, 2018).

### Dopamine and the lead/ethanol interaction in *C. elegans*

Dopamine neurotransmission is related to processes of memory, motivation, reward, locomotion, and addiction, among others (Beaulieu and Gainetdinov, 2011; Koob and Volkow, 2016). Interestingly, *C. elegans* show comparable responses to mammals and other higher organisms regarding substances that affect the DAergic neurotransmission, including the conditioned preference to cues previously associated with drugs of abuse (Lee et al., 2009; Musselman et al., 2012; Katner et al., 2016; Engleman et al., 2018). Thus, alterations in the integrity of this synapse could determine differential responses to neurotoxicants including EtOH, a drug that shares neurobiological mechanisms with other substances promoting DA release in nerve terminals (Söderpalm and Ericson, 2011).

In this line, several reports in higher organisms have provided evidence pointing to the DAergic system as a target of metals, including Pb-induced neurotoxicity (Cory-Slechta and Widzowski, 1991; Pokora et al., 2002; NourEddine et al., 2005). Moreover, multiple DAergic targets sensitive to the toxic action of Pb are simultaneously affected, increasing the vulnerability of this neurotransmitter to Pb toxicity in mammals (White et al., 2007). In *C. elegans* Lu et al. (2018) reported that 60 µM PbCl_2_ administered to adult worms damages the DAergic neurons in 40% of the population, presenting an abnormal phenotype that included alterations in neuronal processes evidenced as a reduction of cell bodies. Moreover, acute treatment with 5 mm Pb acetate to L1 worms induced signs of alterations in almost 80% of the DAergic neurons, accompanied by a reduction in DA levels (Akinyemi et al., 2019). This evidence suggests that alterations in the DAergic neurotransmission are present after both, early-life and adult Pb exposure.

In this regard, results from our laboratory demonstrated that developmental Pb exposure induces morphological alterations in DAergic neurons in a concentration-dependent fashion [0–240 μM Pb (NO_3_)_2_]. In this line, the lowest concentration assessed [24 μm Pb (NO_3_)_2_], although showing minimal alterations in the DAergic synapse, was sufficient to alter the Basal Slowing Response (BSR), a behavior dependent on the integrity of the DAergic system. Interestingly, this response was improved after EtOH (200 mm) only in the Pb-exposed animals that overexpress tyrosine hydroxylase (TH) or are null-mutant of the vesicular transporter (VMAT), whereas the strains lacking the DOP-4 receptor or TH-deficient showed a non-significant-reversal by the drug. These results suggest that EtOH may exert a compensatory effect in the DAergic synapse functional alterations reported in the Pb-exposed animals (Albrecht P. A. et al., 2022).

Furthermore, we have recently demonstrated that control animals treated with 200 mm EtOH reproduced the behavioral phenomenon known as AFT (Davies et al., 2003). Oppositely, perinatally-Pb exposed worms evidenced hyperactivity, which along with a high rate of recovery, was related to impaired EtOH metabolism. To this end, we demonstrated reduced ADH activity as result of early-life Pb exposure. Notably, this effect was not observed in response to 100 mm or 400 mm EtOH, suggesting the requirement of optimal EtOH concentrations for its manifestation. Finally, when another behavior was evaluated, Pb-exposed worms evidenced positive chemotaxis to a site where EtOH was present, revealing the preference of these animals for the drug (Albrecht et al., 2022b, in revision).

### From worms to rats: The Pb and EtOH interaction

**FIGURE 1:**
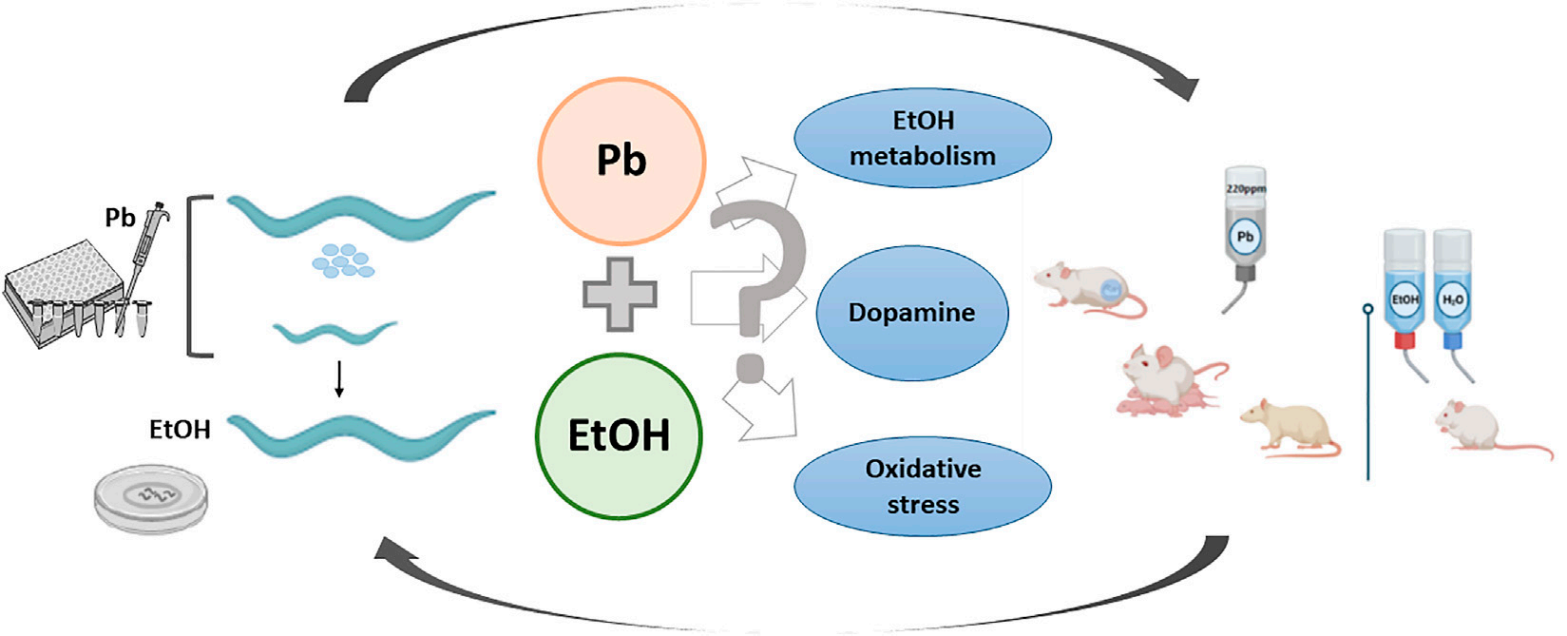
Lead and ethanol exposure protocols in two animal models and possible underlying toxic mechanisms resultant of their interaction.

The above-described stimulant and motivational effects elicited by EtOH in nematodes exposed to Pb during development represent a behavioral phenomenon already described by us in a rodent model. In this regard, Mattalloni et al. reported that 35-day-old Wistar rats perinatally exposed to 220 ppm Pb self-administrated EtOH with a higher break-point than controls. They also consumed more EtOH than their respective controls and presented enhanced locomotor activity after the last voluntary consumption session (Mattalloni et al., 2013; Mattalloni et al., 2017). As with worms, we ascribed these effects to differences in the activity of the enzymes involved in EtOH oxidation (results not shown, reviewed in Virgolini et al., 2017) and their interrelation with oxidative stress (Virgolini et al., 2019), although the participation of the DAergic system was not assessed and thus cannot be discarded. Thus, despite the differences in the experimental design and animal model used in these approaches, we observed in both cases enhanced stimulant and motivational responses to EtOH as a consequence of early-life Pb exposure. These findings raise the possibility of a translational phenomenon from one model to other in the behavioral responses to EtOH. Thus, despite the few limitations of the *C. elegans* model such as the absence of some neurotransmitter systems (noradrenaline) or the scarce evidence regarding others (such as opioids), the results reported here allow us to propose mechanisms of toxicity that may be common for both animal species (Figure 1).

## Conclusions and futures perspectives

The evidence mentioned in this review underpins the usefulness of *C. elegans* in mechanistic studies of environmentally-relevant toxicants such as Pb, even at low exposure concentrations, which may have potential adverse effects later in life. In addition, the revised literature points to this organism as an appropriate tool for a comprehensive phenotypic approach to drugs of abuse, particularly EtOH and associated AUD. This, along with *C. elegans* genetics can be used to evidence the interconnections between different components of behavior and the involvement of environmental toxicants in the modification of drug-induced behaviors from the epigenetic perspective (Scholz and Mustard, 2013; Scholz, 2019). Furthermore, *C. elegans* can be a reliable research platform to test the efficacy of pharmacological compounds used to treat AUDs and the mechanisms of toxicity of environmental contaminants mixtures. Overall, the data provided here and the ample literature on *C. elegans* position this organism in the spotlight as a first-line *in vivo* model to perform exploratory toxicity assessment with potential and accurate escalation to superior animals.
